# Health, mental health, and hearing indigenous voices

**DOI:** 10.1177/10398562241229611

**Published:** 2024-02-07

**Authors:** Andrew Amos

**Affiliations:** Division of Tropical Health and Medicine, College of Medicine and Dentistry, 104397 James Cook University, Townsville, QLD, Australia

**Keywords:** social justice, ethical practice, public health, Indigenous psychiatry

## Abstract

**Objective:**

To identify the issues raised by the unsuccessful Voice referendum and propose removal of barriers to reporting and prevention of family violence in remote communities as the most ambitious measure of success in hearing First Nations voices.

**Conclusions:**

The Voice referendum was partly justified to improve the mental health of First Nations Australians, despite concern the process and its outcome might worsen both. Aboriginal and Torres Strait Islander leaders revealed the tensions that arise between individual and communal interests when marginalised groups fight for self-determination. While a unified First Nation Voice is likely to amplify prominent messengers, we should also be interested in hearing diverse, dissenting voices. As the most marginalised group within a marginalised community, the ability to hear the voices of women and children subject to family violence in rural/remote Australian communities may be the best measure of success in overcoming the barriers that was the motivation for the referendum.

We haven’t had a feminist movement for Aboriginal women because we’ve been expected to toe the line when it’s come to Aboriginal activism for the rights of our race, but the rights as women have been second place…

- Senator Jacinta Price, National Press Club Address, 14.09.23

Those two indigenous people are not ‘progressive No’s’, they are selling out their people, for what reason?

- Professor Pat McGorry, X, 17.09.23

The Uluru Statement from the Heart is a powerful symbol of First Nation Australians' desire for self-determination.^1^ While the RANZCP supported constitutional recognition of Aboriginal and Torres Strait Islanders as likely to improve mental health and wellbeing,^2^ there was also concern that the referendum process and its outcome could have the opposite effect.^3^ Despite the failure of the referendum on 14th October 2023, we should resist the temptation to focus on the loudest Voice or voices while ignoring the diversity of views and interests of First Nations People. This essay does not review the merits of the arguments and experiences described by First Nations leaders during the Voice debate but considers how others should respond. It proposes the visibility of family violence in rural and remote communities as a vital yardstick to measure progress towards a system capable of hearing all First Nations voices.

## First Nations health, mental health, and the need for a voice

There are many gaps between First Nations’ and other Australians' health and wellbeing, widening with remoteness.^4–6^ First Nations Australians report healthcare access requires communication and trust to overcome barriers of distance and logistics.^7^ While we know mental health outcomes are improved by programmes facilitating self-determination, community resilience, and family support, limited information about culturally responsive, accessible care in rural and remote communities significantly limits wellbeing.^6^ Bodies responsible for communicating First Nations’ needs and knowledge to Australian governments are likely to be important in identifying and addressing the causes of disadvantage over the long term.

While most mainstream institutions in Australia supported the call for a Voice to Parliament, there were dissenters. In a speech to the National Press Club on September 14, 2023, Senator Jacinta Price provided the most forceful and articulate expression of the opposition of many Aboriginal people.^8^ Senator Price argued a body comprising Indigenous leaders privileged and shaped by years of access to education and media unimaginable to remote Australians cannot adequately represent their interests.

Senator Price argued that the tendency to treat Indigenous Australians as a single group with common interests contributes to high rates of family violence and sexual abuse suffered by Indigenous women and children. To illustrate, she related a conversation with a senior Indigenous leader who advised her not to report her belief some features of Indigenous culture contribute to family violence. Senator Price understood the advice to reflect both the honest belief of that leader that Indigenous culture does not contribute to family violence, but also that expressing her contrasting belief would harm the interests of Indigenous People as a whole.

Senator Price grounded her argument directly in her own experience and the experience of her mother and many Aboriginal women and children in the communities in which she has lived and represents. She presented as an example the practice of arranged marriages between young women and older men in more traditional Aboriginal communities. Research by the federal government confirms many of Senator Price’s claims, including that family violence increases with remoteness and that it affects all Australians. Contributing factors include social/geographic isolation, stigma/shame, and the lack of privacy when all community members have personal relationships with local police and health professionals.^9^

## Voice or voices?

For an advisory body like the Voice to effectively improve health and mental health it must hear and report all voices, no matter how painful. Senator Price argued that Indigenous people already have multiple voices in the 11 Indigenous representatives elected to the Australian parliament. She described advantages of elected representatives responsible to both Indigenous and non-Indigenous constituents over a racially homogenous Voice. Such representatives are required to integrate the different interests of diverse people, which is likely to improve their ability to communicate with and convince all Australians.

Nevertheless, some leaders want to prevent Australians from hearing Senator Price. Prominent Voice advocate Professor Marcia Langton has said that ‘Jacinta Price is useful to politicians. She legitimises racist views by speaking them against her own people…exploiting her mother [Bess Price]’s brand of anti-violence campaigning and appeal to the scientific racism of the alt-right’.^10^ Even more extreme, former Australian of the Year Professor Pat McGorry has accused Senator Price and Warren Mundine of ‘selling out their people’.^11^ In common with Professor Langton, Professor McGorry’s statement denies the reality of the violence suffered by Senator Price, her mother, and her people. It implies that Senator Price’s actions are calculated to achieve power and material success, and endorses existing power structures which Senator Price criticises, and in which both Professor Langton and Professor McGorry have an interest.

## Comparing voice and visions

A key point in the Voice debate was whether the final form of the Voice should have been settled before calling a referendum.^12^ Without relitigating the arguments, it appears incontestable that the lack of a defined model made it impossible to judge what sort of impact the Voice might have had on First Nations and other Australians.

In response to the question how she would address Indigenous Australians’ health and wellbeing, Senator Price said that she wanted an investigation of all government funded schemes targeting First Nations welfare, as she believed existing programmes failed to achieve their goals because leaders were unaccountable.^8^

While it is for First Nations leaders to choose their approach to self-determination, others must consider the implications for government and health systems, including mental health. From this point of view, the referendum result may be less important than the concerns raised. It is difficult to deny that government responses would benefit from detailed understanding of the experience of the broad range of First Nations Peoples across Australia. It seems equally difficult to argue against the ideas that some voices have not been well represented in existing frameworks, and that leaders must be responsible for the initiatives they champion.

## Hearing secret harmonies

As perhaps the most recognisable Voice advocate, Noel Pearson also appears to have most successfully integrated Professor Langton’s unapologetic demand for self-determination with Senator Price’s call for accountability.^13^ He has criticised both the imposition of external control on First Nations People and the ‘soft bigotry of low expectations’ that arises when the earnest desire to avoid ‘blaming the victim’ prevents a clear understanding of the factors that cause and maintain disadvantage.^14^

Despite the referendum result, strong, independent First Nations voices will continue to assert their right to be heard. It is unrealistic to expect that all First Nations People will agree on all or even most things. If it is accepted there will be disagreements, then it is vital not to exclude voices from the debate because they say things that we do not agree with. It is particularly important that we do not accuse those with different opinions and different experiences of bad faith. Rather, we should accept they are making the best case they can to achieve the common goal of eliminating disadvantage.

While First Nations People have the right to choose their own representatives and pursue their own goals, they must engage with the same government, health, and welfare systems that support all Australians. While there have been recent improvements, such as increased access to inpatient mental health care, it is clear that First Nations Australians in rural and remote locations are often less visible to these systems than others, preventing early detection and timely intervention.^4,6,9^

The suggestion that the health and mental health of First Nations women and children in rural and remote communities is affected by cultural factors is difficult to discuss. However, any process likely to improve the mental health and wellbeing of all First Nations People must be able to mediate this type of discussion. When Senator Price, or one of the Aboriginal women she referred to in her speech, speaks out about their experiences in remote communities, the capacity to investigate, understand, and report what has happened must exist. A political system that does not hear Jacinta Price and attempt to understand her experience cannot adequately represent her and those who share her experiences.

## Conclusions

The Uluru Statement from the Heart and the referendum offering a Voice to Parliament to the Australian people were loud expressions of an Aboriginal and Torres Strait Islander Voice comprising many individual voices. Support for the Voice was justified in part by the hope that it would improve the mental health and wellbeing of First Nations People. While it is for First Nations People to determine how they communicate as a community or communities in the wake of the referendum’s defeat, those listening should want to hear all voices. The success of alternatives to the Voice should be measured by how well they engage with the most difficult questions, such as the role of cultural factors in the health and mental health of women and children in remote communities.
